# Isolated Lung Perfusion as an Adjuvant Treatment of Colorectal Cancer Lung Metastases: A Preclinical Study in a Pig Model

**DOI:** 10.1371/journal.pone.0059485

**Published:** 2013-03-18

**Authors:** Pierre-Benoit Pagès, Olivier Facy, Pierre Mordant, Sylvain Ladoire, Guy Magnin, Francois Lokiec, Francois Ghiringhelli, Alain Bernard

**Affiliations:** 1 INSERM UMR 866, CHU Bocage, University of Burgundy, Dijon, France; 2 Department of Thoracic and Cardiovascular Surgery, CHU Bocage, University of Burgundy, Dijon, France; 3 Department of Digestive Surgery, CHU Bocage, University of Burgundy, Dijon, France; 4 Department of Thoracic Surgery, Georges-Pompidou European Hospital, Paris, France; 5 Department of Medical Oncology, Georges-Francois Leclerc Center, University of Burgundy, Dijon, France; 6 Department of Anesthesiology, CHU Bocage, University of Burgundy, Dijon, France; 7 Department of Pharmacology, Centre René Huguenin, Saint-Cloud, France; Baylor University Medical Center, United States of America

## Abstract

**Background:**

The lung is a frequent site of colorectal cancer (CRC) metastases. After surgical resection, lung metastases recurrences have been related to the presence of micrometastases, potentially accessible to a high dose chemotherapy administered via adjuvant isolated lung perfusion (ILP). We sought to determine *in vitro* the most efficient drug when administered to CRC cell lines during a short exposure and *in vivo* its immediate and delayed tolerance when administered via ILP.

**Methods:**

First, efficacy of various cytotoxic molecules against a panel of human CRC cell lines was tested *in vitro* using cytotoxic assay after a 30-minute exposure. Then, early (operative) and delayed (1 month) tolerance of two concentrations of the molecule administered via ILP was tested on 19 adult pigs using hemodynamic, biological and histological criteria.

**Results:**

*In vitro*, gemcitabine (GEM) was the most efficient drug against selected CRC cell lines. *In vivo*, GEM was administered via ILP at regular (20 µg/ml) or high (100 µg/ml) concentrations. GEM administration was associated with transient and dose-dependant pulmonary vasoconstriction, leading to a voluntary decrease in pump inflow in order to maintain a stable pulmonary artery pressure. After this modulation, ILP using GEM was not associated with any systemic leak, systemic damage, and acute or delayed histological pulmonary toxicity. Pharmacokinetics studies revealed dose-dependant uptake associated with heterogenous distribution of the molecule into the lung parenchyma, and persistent cytotoxicity of venous effluent.

**Conclusions:**

GEM is effective against CRC cells even after a short exposure. ILP with GEM is a safe and reproducible technique.

## Introduction

About 11% of patients suffering from colorectal cancer (CRC) will eventually develop lung metastases [1]. This condition is associated with 5-year survival rates ranging from 5 to 50%, according to the possibility to achieve complete surgical resection [2]–[7]. Despite surgical resection, pulmonary recurrence occurs in more than 40% of patients, probably due to micrometastases that were already present during the initial procedure [3]. Adjuvant chemotherapy improves progression-free but not overall survival [8], and the increase of systemic dose is associated with unacceptable toxicities [9].

The complete tumor and metastasis control has always been the cornerstone of anticancer therapies. Furthermore, recent data indicate that metastasis is a multidirectional process whereby cancer cells can seed distant sites as well as the primary tumor itself [10]. This later process, known as 'self-seeding,' has been validated in diverse experimental models [11], and constitute a strong argument in favour of the local control of metastatic diseases.

In order to decrease the recurrence of CRC lung metastases, some authors have developed the technique of isolated lung perfusion (ILP) [12], which permits to increase the dose of cytotoxic agent delivered to the lung tissue without systemic exposure. This technique has been proven to be safe and effective against CRC lung metastases in rodent model [13]–[17]. The main objectives of this study were to determine *in vitro* the most efficient drug when administered to CRC cell lines during a short exposure and *in vivo* its immediate and delayed tolerance when administered via ILP in a pig model.

## Materials and Methods

### In vitro Anti Tumoral Effect

#### Cell lines

A panel of human colorectal adenocarcinoma cell lines (HT29, HCT8, HCT116, SW480) was chosen to represent the diversity of CRC chemo sensitivity and mutational status (Table 1), purchased from the American Tissue and Cell Collection (ATCC®, Rockville, MD, USA) and maintained in culture as recommended.

**Table 1:** Mutation status of CRC cell lines tested in vitro.

Cell line	MutantK-RAS Ex 2	MutantBRAF exon 15	Mutant PIK3CA exon 9	Mutant PIK3CA exon 20	MSI Status	MSS Status
HT29		+				+
HCT8	+		+		+	
SW480	+					+
HCT116	+			+	+	

#### Compounds

The following compounds were chosen as the most efficient drugs against CRC in the clinical setting [18]–[19]: Gemcitabine (GEM) (Gemzar®, Lilly), Cisplatin (Cisplatine Mylan®, Mylan), 5 Fluorouracil (5 FU) (Fluorouracile Accord®, Accord Healthcare), Oxaliplatin (Eloxatine®, Sanofi), Raltritexed (Tomudex®, Hospira) and Irinotecan (Campto®, Pfizer).

#### Cytotoxicity assay

Anti tumoral effect of these compounds was determined using cytotoxic assay, as previously described [20]. Briefly, cells were seeded in 24-well plates, allowed to grow until confluence in 24 hours, and exposed to selected compounds alone or in combination for 30 minutes. After 4 days, cells were stained using crystal violet, and absorbance was measured in each well by an automatic photometer (590-nm filter, Spectra-A®, Varian, France). Cell survival was calculated as the percentage of absorbance in treated *vs* untreated wells, and IC50 (concentration achieving an inhibition of growth of 50% of cells) were determined. The most efficient drug was defined as the compound with the lowest IC50 [20], and tested in combination with the second most efficient compound.

### Isolated Lung Perfusion

#### Animals

Three-month old large white pigs (n = 19), weighing 50±3 kg each, were purchased from Hazotte (Beaumont, France). Animals were allowed to acclimatize to the laboratory environment for 7 days with free access to food and water. Experiments were approved by the Animal Ethics Committee of the University of Burgundy, France (A0809).

#### Anesthesia

Animals were anaesthetized as previously described [21]. The systemic arterial blood pressure was monitored through a catheter inserted into the humeral artery. The heart rate, electrocardiogram, nasal temperature, oxygen blood saturation were monitored using the NICO system (Novametrix Medical Systems, Wallingford, CT). Unfractionated heparin (100 UI/kg) was administered before vascular exclusion of the lung. To achieve perioperative analgesia, 20 mL of ropivacaine 0.75% were injected into the perilesional skin and chest wall. Tramadol and paracetamol were prescribed in the postoperative period at regular intervals.

#### Surgical Technique

Having placed the animal on right lateral decubitus, a left postero-lateral thoracotomy was performed in the fourth intercostals space. Pericardium was opened widely, and the left main pulmonary artery (LMPA) and both left pulmonary veins (LPV) were isolated. Cannulation was performed using a metal tipped right-anguled cannula (High Flow Aortic Arch Canula 3.8 mm, Terumo®, Ann Arbor, USA) inserted into the LMPA and a venous cannula (DLP Left Heart Vent Catheter, Medtronic®, Minneapolis, USA) inserted into the convergence of the LPVs via the left atrium (LA). A monitor line was inserted into the origin of the LMPA. The LMPA and LA were than clamped, the left lung was ventilated and the left main bronchus was snared to occlude bronchial arterial blood [22].

#### Isolated Lung Perfusion

The extracorporeal circulation system comprised a pump (Biomedicus®, Minneapolis, USA), heat exchanger, reservoir and PVC tubing of ¼ inch diameter. Priming was achieved with a solution containing 850 ml of voluven (6% hydroxyethyl starch 130/0.4) and 150 ml of blood from the lung circulation. After starting the perfusion, the pump flow was gradually increased to obtain a mean LMPA pressure equivalent to the pressure measured before clamping. Chemotherapy was then injected into the circuit [23], pump flow was modulated to stabilize mean LMPA pressure, and chemotherapy perfusion lasted for 30 min followed by a 15 min period of washout. At 5, 10, 20 and 30 min of the perfusion, systemic blood samples were taken. At 30 min of perfusion, two lung samples were taken to measure the concentration of drug in the lung tissue and to assess the histological acute lung injury. Fluid samples were taken in the perfusion circuit to measure the concentration of the drug, and lung effluent was drawn to evaluate its cytotoxic effect on tumor cells *in vitro*. At the end of the procedure, cannulas were withdrawn, thoracotomy was closed on a temporary chest tube, and animals were awakened.

#### Experimental groups

Phase I study has defined a maximum tolerated dose following a 30-minute IV infusion of GEM equivalent to systemic dose of 20 µg/ml/h [24]. As a projection, the concentration of GEM used for ILP was 0 µg/ml (control group, n = 5), 20 µg/ml (regular dose group, n = 7 animals) and 100 µg/ml (high dose group, n = 7).

#### Pharmacokinetics

To determinate the concentration of GEM into blood samples and lung parenchyma, samples were spin-dried at 4°C and 12000 G during 10 minutes. Quantification of GEM in the plasma and surpernatant was then achieved using isocatic reverse-phase high-performance liquid chromatography (HPLC) system as previously described [25]. Concentration of GEM at the end of the ILP was established in the circuit, in two different lung biopsies, and in the systemic circulation. Residual cytotoxicity of the venous effluent was studied using serial dilutions and in vitro cytotoxic assay.

#### Acute systemic toxicity

The ILP procedure was performed on nineteen pigs. Systemic toxicity of GEM was evaluated by measurement of liver and renal function. Hemodynamic tolerance was assessed by recording heart rate and systemic blood pressure.

#### Acute pulmonary toxicity

Respiratory tolerance was assessed by recording mean LMPA pressure, oxyhaemoglobin saturation (SpO2) and partial pressure of carbon dioxide during expiration (PCO2). The severity of lung lesions was assessed histologically using the Chiang score, which was created to evaluate acute lung injury after the ischemia/reperfusion sequence of transplanted lungs [26]. Briefly, this score esteems lesions as edema and cell infiltration. Edema is scored as 1 for perivascular edema; 2 for peribronchial or interstitial edema; 3 for alveolar edema. Cell infiltration is scored as 2 for perivascular cell infiltration; 3 for interstitial cell infiltration; 4 for alveolar cell infiltration. The sum of all the pathological scores was the score for each scope, and then we calculated the mean score for the left lung by the sum of the upper and the lower lobe score, with 0 being a normal score.

#### Delayed toxicity

After one month of follow-up, twelve animals were reoperated on. Delayed toxicity was assessed using the same methods and criteria as described for acute toxicity. Animals were then humanely killed by an intracardiac injection of Pentobarbital (Dolethal®, Vetoquinol).

#### Statistical analysis

Categorical variables were reported as numbers and proportions. Normal continuous variables are reported as means ± standard deviations. Concerning the hemodynamic parameters, we compared the moderate mean values of a way repeated to the same animal by repeated-measures ANOVA. A linear regression was performed to estimate the correlation between continuous variables. When the results of the variance analysis were statistically significant (p<0.05), we performed a *posthoc* comparison using Benferroni’s method to reduce the α risk. Statistical analyses were performed using the STATA 12 statistical software (StataCorp, College Station, USA).

## Results

### Cytotoxic Assay

**Figure 1:**
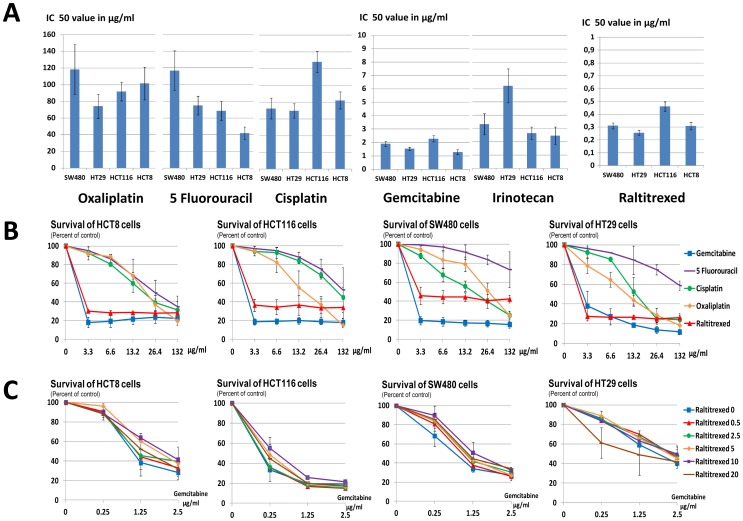
A- IC50 values after in vitro exposure for 30 min of CRC cells SW480, HT28, HCT116 and HCT8 with Oxaliplatin, 5 FU, Cisplatin, GEM, Irinotecan and Raltitrexed. B- Dose-response curves after in vitro exposure for 30 min of CRC cells SW480, HT28, HCT116 and HCT8 with Oxaliplatin, 5 FU, Cisplatin, GEM, Irinotecan and Raltitrexed. C- Dose-response curves after in vitro exposure for 30 min of CRC cells SW480, HT28, HCT116 and HCT8 with combination of GEM and Raltitrexed.

Administered through a short exposure, GEM was more efficient than 5 FU, cisplatin, oxaliplatin and irinotecan (Figure 1 A & B). Raltitrexed showed the same efficacy as GEM in HCT8 and HT29, but not in HCT116 and SW480 cells. Adjunction of raltitrexed to GEM did not increase its cytotoxicity (Figure 1 C). Therefore, GEM alone was selected for the ILP procedure.

### Pharmacokinetics

**Figure 2:**
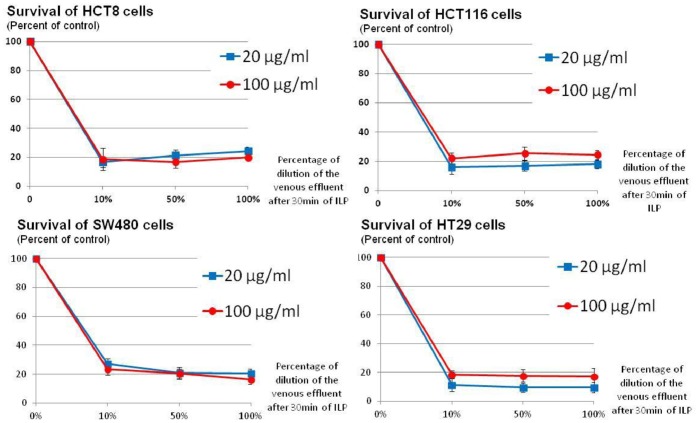
Dose-response curves after in vitro exposure for 30 min of CRC cells SW480, HT28, HCT116 and HCT8 with the lung effluent collected at the end of the ILP procedure. Cells were exposed to different dilutions (10%, 50% and 100%) of the lung effluent.

After 30 min of perfusion, GEM concentration was greater for the high-dose than for the regular-dose group when measured in the circuit (74.29±18.06 vs 22.85±6.51 µg/ml respectively, p = 0.0001) and in the lung parenchyma (141.46±117.07 µg/g vs 56.91±28.06 µg/g respectively, p = 0.014). Cytotoxicity assay showed a maintained cytotoxic effect of the lung effluent at the end of ILP, with no difference between the regular-dose and the high-dose group even for the 10% dilution of the lung effluent (Figure 2). In the systemic circulation, the maximum concentration of GEM after ILP was 0.638±0.27 µg/ml for the high-dose group and 0.248±0.15 µg/ml the regular-dose group (Table 2).

**Table 2:** Maximun GEM leaks in the systemic circulation during the ILP procedure (combination of 20 and 100 µg/ml groups, n = 14).

Time of perfusion (min)	Gemcitabine leaks in the systemic circulation	
	20 µg/ml	100 µg/ml
0	0	0
5	0.248±0.15	0.638±0.27
10	0.219±0.11	0.598±0.23
20	0.212±0.1	0.540±0.21
30	0.208±0.11	0.510±0.17

### Acute Systemic Toxicity

Regarding systemic hemodynamic tolerance, no significant change was observed in blood pressure, heart rate, and electrocardiogram as determined before, during, and after the procedure (Table 3). No major liver or renal acute toxicity was noted (Table 4).

**Table 3:** Hemodynamic and respiratory parameters during ILP procedure.

Hemodynamic and respiratory parameters studied	Before ILP	During ILP	After ILP	P-value
Systolic Blood Pressure (mmHg)	97.60±13.56	95.226±16.07	91.333±17.94	0.5585
Diastolic Blood Pressure (mmHg)	49.8±16,47	49.8±15.13	44.466±15.75	0.57
Heart Rate (/min)	102.26±22.2	107.33±19.6	104.33±21.2	0.8035
SpO2 (%)	98.6±1.72	98.266±2.05	98.33±1.71	0.8714
PCO2 (mmHg)	36.66±7.41	34.13±7.09	35.66±7.63	0.642

**Table 4:** Renal and hepatic functions of the animals at the end of the ILP and after one month of survival.

	Gemcitabine concentration in lung perfusate (µg/ml)	Biological parameters			
		ALAT (U/L)	ASAT (U/L)	Alk Ph (U/L)	Creat (mg/L)
Preoperative mean values		30±7	50±11	108±20	14.2±3.4
At the end of the ILP procedure	0	38±19	68.33±54.3	87.33±15.5	17.56±0.66
	20	53.2±39.62	69±64.07	103.2±21.95	13.03±2.39
	100	23.75±7.54	30.25±7.63	115.75±15.08	14.27±4.92
After one month of survival	0	25.33±3.21	30.35±7.63	115.75±15.08	14.27±3.48
	20	24±4.12	23±8.86	114.4±21.81	16.56±4.14
	100	27.25±4.34	26.5±12.39	126.25±18.11	16.56±3.87

*ALAT* alanine aminotransferase, *Alk Ph* alkaline phosphatase, *ASAT* aspartate aminotransferase, *Creat* creatinemia.

### Acute Pulmonary Toxicity

**Figure 3:**
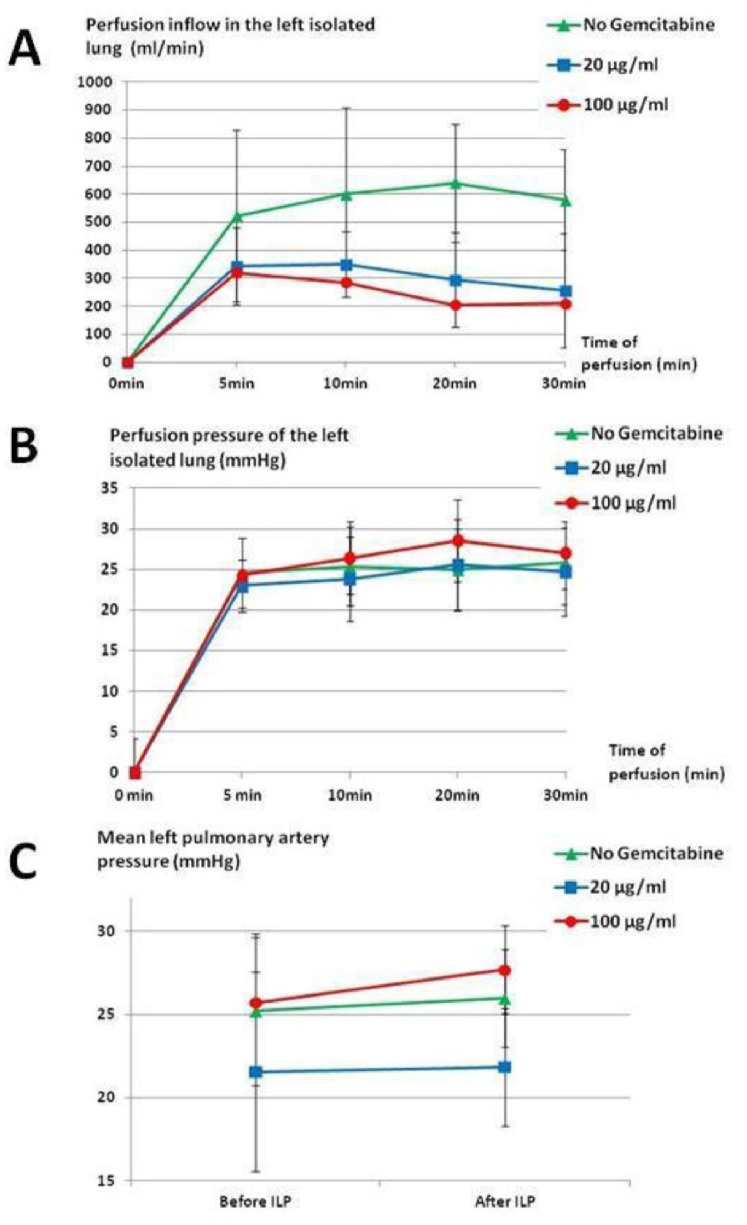
A- Perfusion inflow in the isolated lung perfused with gelatin (580±178.88 ml/min), GEM 20 µg/ml (257.14±202.62 ml/min) and GEM 100 µg/ml (210+/−138.92 ml/min) during 30 min (p = 0.0055). B- Perfusion pressure in the isolated lung perfused with gelatin, GEM 20 µg/ml, and GEM 100 µg/ml during 30 min. C- Mean left pulmonary artery pressure before and after ILP with gelatin, GEM 20 µg/ml and GEM 100 µg/ml during 30 min (p = 0.7187).

Before clamping, individual variations in the mean LMPA pressure was noted at baseline, with values ranging from 18 to 34 mmHg. After clamping, the perfusion inflow was progressively increased to achieve an inflow of 500 to 600 ml/min. In the GEM groups, the mean LMPA pressure increased gradually, leading to decrease the inflow of the extracorporeal pump to maintain the initial mean LMPA pressure (Figure 3A). As a consequence, the mean LMPA pressure was not significantly different between the three groups (Figure 3B), but the mean perfusion inflow at 30 min was lower in the GEM groups than in the control group (Figure 3A). There was an inverse correlation between the concentration of GEM in the perfusion circuit and the average flow after 30 min of perfusion (p = 0.0158). There was no difference in the mean LMPA pressure before and after ILP (p = 0.7187, Figure 3C). Histological analyses revealed a mean Chiang score of 5.05±3.52 in the control group, with no significant difference in the GEM groups. There was also no correlation between the Chiang score and the GEM dose infused (p = 0.7491, Table 5) or the GEM concentration into the lung parenchyma (p = 0.7278).

**Table 5:** Chiang scores at the end of the ILP procedure and one month later.

	Concentration of Gemcitabine in the perfusate (µg/ml)	Mean acute lung injury score of Chiang for the left lung	P-value
At the end of the ILP procedure	0	5.05±3.52	0.7491
	20	4.14±3.10	
	100	5.46±3.28	
After one month of survival	0	10±2.29	0.0846
	20	6±1.66	
	100	6.12±1.94	

### Long Term Toxicity

Neither lung necrosis nor fibrosis was observed, either macroscopically or microscopically. Most frequently lesions described were peribronchial edema and interstitial cell infiltration. Severe lesions such as alveolar cellular infiltration were only seen for the three first animals in the control group, who presented a pulmonary edema at the end of the ILP procedure related to a rapid increase of the inflow. There was no correlation between the GEM concentration in the lung parenchyma and the Chiang score (p = 0.0846) (Table 5).

## Comments

Initially developed as an adjuvant treatment of lung metastases originating from sarcomas, ILP has generated considerable interest because of the high pulmonary recurrence rate and decreased long-term survival in this pathology, even after complete surgical resection [3]. Potential advantages of ILP include the delivery of high-dose chemotherapy to the lung, leading to rapid saturation of the lung parenchyma and limited systemic leak [14], [27]–[29].

The interest of the *in vitro* cytotoxic assay is to choose a chemotherapy drug for the ILP procedure, as suggested by previous studies. Link *et al*. demonstrated that *in vitro* assays could identify active drugs for individualized hepatic artery infusion [30]. Kornmann *et al*. showed that patients with CRC that demonstrated *in vitro* sensitivity may benefit from pancreatic and colic regional chemotherapy with GEM [31]. When setting this preliminary assay, we chose a panel of cells representing the diversity of CRC concerning growth factors signalling pathways [32]. These pathways are targeted in case of metastatic disease, alone or in combination with cytotoxic agents including oxaliplatin and irinotecan [33]. However, these two drugs were less efficient than GEM following short exposure, probably because GEM exhibits a dose-dependent effect whereas the effect of oxaliplatin and irinotecan is more time-dependent [24], [34], [35].

To date, GEM is not a standard systemic treatment of metastastic CRC [33]. However, as previously stated, some publications justify the use of the HTCA to choose the active chemotherapy on CCR cells during a short exposure of 30 min [30], [31]. It is to note that drugs used in isolated organ perfusion, such as isolated lung or liver perfusion, are not necessarily the molecules used for intravenous treatments. In thoracic oncology, Hendricks et al. have reported encouraging results in a phase I clinical trial using melphalan during ILP to treat lung metastases [22]. In this study, primary cancers were colorectal carcinoma, renal cell carcinoma and sarcoma, and melphalan was not a standard therapy of any of these cancers. Similarly, in hepatic oncology, melphalan has been reported to be associated with clinical response and prolonged survival following isolated liver perfusion, despite its limited efficacy following intra venous administration [28]. Isolated organ perfusion remains an experimental technique performed only by few surgical teams in the clinic, and all the drugs available for systemic administration have not been tested for isolated organ perfusion. One of the main advantages of isolated organ perfusion is to allow the use of higher dose of cytotoxic agents, thus expanding the number of molecules available to obtain an antitumoral effect. However, to date, there is no publication in evaluating the efficacy of gemcitabine on lung metastases from CRC in the clinical setting.

Regarding isolated organ perfusion, the classical dose escalation design is useless if extracorporeal circuit lead to a stable state exposing the organ parenchyma to active concentrations associated with normal tissue saturation [36]. In our study, GEM concentrations used during ILP were eighteen to forty-fold higher than *in vitro* IC50, leading to stable GEM concentrations in the circuit throughout the perfusion, with minimal systemic leaks. For both groups, GEM concentrations in the circuit were much higher (from three to twenty-fold) to that found in the plasma of patients treated with IV GEM [24]. Furthermore, the cytotoxic activity of lung effluent demonstrated the persistence of high GEM concentrations and preserved cytotoxic activity. These three elements indicate a saturation of the lung parenchyma as a whole by GEM during the ILP procedure, but spatial distribution remains doubtful.

During ILP, the inflow pressure was maintained equivalent to the mean pulmonary arterial pressure measured before clamping to prevent pulmonary edema and acute lung injury [37], [38]. Adjunction of GEM to the circuit was associated with an increase of intrapulmonary vascular resistance and subsequent increase of the mean pulmonary artery pressure (mPAP). This transient vascular toxicity of GEM has already been reported following systemic administration, and described as a transient vasoconstriction associated with a capillary leak syndrome [39]. We showed that during ILP this vascular phenomenon is dose-dependent and reversible, providing that lowering the ILP inflow controls the mPAP. Finally, we found heterogeneous concentrations of GEM in the lung parenchyma according to the region and whatever the group considered, as already reported with other cytotoxic agents [40]. This heterogeneity can be interpreted as a consequence of a patchy drug-induced vasoconstriction, and may be counterbalanced by the adjunction of vasodilatators during ILP using GEM.

Higher histological Chiang score were found in the control group than in the GEM group, at the end of the procedure and after one month of survival. Franke et al. have demonstrated that increased pulmonary pressure in a setting of ILP will result in deleterious effects on morphology [37], but in our study, the pressure of perfusion was significantly different in the control and GEM groups. The only significant difference in pulmonary hemodynamic between groups was a significant higher perfusion inflow in the control group. This increase of the perfusion inflow may be responsible of the histological deleterious effects.

This study demonstrated that ILP with GEM is a safe and reproducible technique, permitting to deliver high dose of chemotherapy to the lung parenchyma with very limited systemic leaks. However, GEM-associated transient and dose-dependant pulmonary vasoconstriction led to heterogeneous distribution of GEM in the lung parenchyma. Further experimentations are needed to optimize the distribution of the drug into the lung parenchyma and to test the efficacy of this approach before considering clinical studies.
